# TUDCA combined with Syndopa protects the midbrain and gut from MPTP toxicity in a Parkinson’s disease mouse model: Immunohistochemical evidence

**DOI:** 10.17305/bb.2025.12519

**Published:** 2025-08-04

**Authors:** Mahalakshmi Rajan, Senthilkumar Sivanesan, Kalpana Ramachandran, Pankaj Goyal, Priya Palanivelu, Anamitra Ghosh, Rajagopalan Vijayaraghavan, Smitha S Vasavan

**Affiliations:** 1 Department of Research and Development, Saveetha Institute of Medical and Technical Sciences, Chennai, India; 2 Department of Anatomy, Sri Muthukumaran Medical College and Research Institute, Chennai, India; 3 Department of Anatomy, Sri Ramachandra Medical College and Research Institute, Chennai, India; 4 Department of Biotechnology, Central University of Rajasthan, Ajmer, India; 5 Department of Anatomy, KMCH Institute of Health Sciences and Research, Coimbatore, India; 6 Baylor College of Medicine, Houston, TX, USA; 7 Department of Anatomy, PMS College of Dental Science and Research, Thiruvananthapuram, India

**Keywords:** Parkinson’s disease, PD, MPTP, TUDCA, Syndopa, α-synuclein, substantia nigra

## Abstract

Neuro-inflammation plays a significant role in the neurodegenerative processes associated with Parkinson’s disease (PD). A hallmark of PD is the degeneration of dopaminergic neurons within the nigrostriatal pathway. The standard treatment for PD is Syndopa (a combination of levodopa and carbidopa). However, while Syndopa alleviates symptoms, it is also associated with numerous side effects in patients. Research has demonstrated the protective effects of Tauroursodeoxycholic acid (TUDCA) in mitigating the neuropathological consequences of PD in several preclinical studies. Nonetheless, further investigation is necessary to delineate the role of TUDCA in PD therapeutics. Although the efficacy of TUDCA monotherapy in PD has been explored, there is a lack of preclinical research examining the additive effects of TUDCA in conjunction with Syndopa therapy. In this study, we utilized a 1-methyl-4-phenyl-1,2,3,6-tetrahydropyridine (MPTP) mouse model of PD to evaluate the potential therapeutic benefits of TUDCA monotherapy and the combined effects of TUDCA and Syndopa therapy, compared to standard Syndopa treatment. We conducted immunohistochemical (IHC) assessments of α-synuclein expression in the gut and substantia nigra pars compacta (SNpc), as well as tyrosine hydroxylase (TH) and NF-kB expression in the striatum and SNpc regions, to investigate the efficacy of the test drugs. The IHC findings indicate that both TUDCA monotherapy and the combination therapy of TUDCA and Syndopa significantly reduced MPTP-induced alterations in the expression levels of α-synuclein, TH, and NFκB in the striatum and SNpc regions. Additionally, the MPTP-induced changes in α-synuclein expression in the gut were notably reversed by these treatments. Collectively, these results suggest that incorporating TUDCA with Syndopa may represent a promising therapeutic strategy to address the pathophysiological challenges associated with PD.

## Introduction

Parkinson’s disease (PD), often referred to as paralysis agitans, is a degenerative brain condition that affects the central nervous system’s extrapyramidal motor neurons. It is one of the detrimental neurodegenerative conditions in the elderly, next to Alzheimer’s disease (AD) [1]. James Parkinson was the first to describe the cardinal manifestations of PD. Pathologically, it is classified as a synucleinopathy, along with other conditions that have Lewy bodies [2]. It is predicted to follow dopaminergic neuronal death in the substantia nigra pars compacta (SNpc), depleted striatal dopamine levels, and proteinaceous clumps within neuronal cell bodies called Lewy bodies, which stain for α-synuclein [3]. Neuronal degeneration with Lewy bodies is not only restricted to the dopaminergic system; it also affects the non-dopaminergic neuronal system and the peripheral nervous system [4].

About 90% of cases of PD occur sporadically and are due to unknown etiology. The remaining 10% of cases are familial, occurring due to mutations in the *SNCA*, *GBA*, *LRRK2*, and *PINK1/PRKN* genes [5, 6]. Although the exact cellular mechanisms contributing to dopaminergic degeneration in PD are ambiguous, it is believed that oxidative stress, neuroinflammation, and mitochondrial dysfunction play a major role in the depletion of dopaminergic cells in sporadic and familial PD [7, 8]. Glial cells, which have a significant role in antioxidant defense [3], are known to release several pro-inflammatory mediators and phagocytose cellular debris when activated by dying neurons [7, 9, 10]. This further contributes to the degeneration of neurons, with provoked inflammation and cell death that aggravates and intensifies the neurodegenerative process [11]. In fact, glial activation contributes significantly to the development of neuronal dysfunction in PD.

Clinically, PD is manifested by motor symptoms such as resting tremor, bradykinesia, muscle rigidity, and postural imbalance [12]. In addition to the above-mentioned “cardinal” motor symptoms, PD also manifests in the form of other non-motor symptoms that include dysphagia, speech problems, constipation, anxiety, depression, orthostatic hypotension, micturition abnormalities, and cognitive problems. Several of these are recognized as prodromal symptoms of PD or risk factors for the illness [13, 14].

In PD patients, the caudate nucleus and putamen become excessively active and continuously produce excitatory signals to the corticospinal tract due to the destruction of dopaminergic neurons, which causes rigidity. Dopamine is the inhibitory transmitter released in these regions. Levodopa, or L-dopa (LD), a precursor of dopamine, is a commonly used treatment drug for PD symptoms. However, there are certain harmful effects associated with LD therapy, such as hypotension, nausea, gastrointestinal hemorrhage, sleeplessness, disorientation, and auditory hallucinations [15].

1-Methyl 4-phenyl 1,2,3,6-tetrahydropyridine (MPTP) is used for developing PD in animal models, which reproduce the neurodegeneration. MPTP, being a lipophilic component, easily crosses the blood–brain barrier (BBB) and combines with astrocytes, where it is metabolized by monoamine oxidase (MAO) into its active metabolite MPP+ [16, 17]. MPP+ has tropism for dopaminergic neurons and is actively taken up by the DA neurons, where it is believed to cause neurodegeneration in those neurons by inducing mitochondrial dysfunction, oxidative stress, inflammation, excitotoxicity, and aggregation of inclusion bodies [18–21].

LD, a commonly used drug to relieve PD symptoms, poses many adverse responses. The episodes of various motor symptoms due to dopa resistance and non-motor symptoms, which encompass autonomic dysfunctions, mood and cognitive impairment, and drug-linked side effects (psychosis, motor fluctuations, and dyskinesia), are notable features [22, 23]. Owing to the longer half-life of dopamine agonists, they are increasingly being used in the treatment of PD as monotherapy or as a combination drug [24]. The present research scenario focuses on identifying an effective treatment that will be better than LD or on implementing pharmacological approaches to reduce the LD dose levels to prevent side effects. Although α-synuclein accumulation seems to play a major role in PD and is considered one of the most supported hypotheses among several, the exact pathogenic mechanisms of PD are still unclear and warrant more detailed investigations. The course of PD involves several events, which include mitochondrial dysfunction, neuroinflammation, and faulty protein clearance mechanisms [19]. However, how these various events cooperate and integrate with each other remains incompletely understood [25]. While this movement disorder occurs due to the damage of dopaminergic neurons in the SNpc, other brain regions are also critically affected [11, 26] and extensive investigations are underway. Several seminal works have indicated the importance and significance of gut dysfunctions in PD pathogenesis. Although the LBs are identified as pathological hallmarks of PD, containing mostly the aggregated α-synuclein protein, how they lead to neurodegeneration is still obscure.

Tauroursodeoxycholic acid (TUDCA), a natural bile acid, is an endogenous taurine conjugate of ursodeoxycholic acid (UDCA) that is employed in the management of cholestatic liver diseases. It crosses the BBB and is found to be nontoxic. Research has been conducted widely to understand the beneficial role of TUDCA in many non-liver diseases, such as neurodegenerative diseases. In fact, TUDCA has shown a considerable neuroprotective role in mouse models of Alzheimer’s and Huntington’s diseases [27–30].

The proposed mechanisms of TUDCA’s neuroprotective activity in an experimental model of PD include activation of the pro-survival Ser/Thr kinase Akt and anti-oxidative mechanisms dependent on the nuclear factor E2-related factor 2 (Nrf2) pathway, as well as parkin-mediated mitochondrial turnover [31–34]. However, studies that support the gut protective mechanisms of TUDCA in PD have not been found.

Brain histomorphological studies can be used to delineate TUDCA’s neuroprotective role and efficacy in PD. So far, the therapeutic potential of TUDCA has been tested as monotherapy in PD preclinical research. In the present work, we tested TUDCA’s pharmacological potential in MPTP-intoxicated mice through immunohistochemical studies involving brain and gut samples. Notably, in the present work, TUDCA was tested both alone and as an additional drug along with Syndopa to explore the neuroprotective benefits.

## Materials and methods

### Animals

The Mass Biotech animal facility in Chengalpattu, India, supplied C57BL/6 mice (male), which were 2–3 months old and weighed 30–40 g. The mice were allowed 7 days for acclimatization, followed by 26 days of experimentation. Mice were kept in a clean environment with a relative humidity of 30%–40% and a photoperiod of 12 h of light and 12 h of darkness. Throughout the investigation, the mice had unrestricted access to food and water.

### Mice grouping and experimental protocol

The mice were randomly segregated into five groups, each having six mice, as described below: The randomization of animals was done using the Random Number Table method. Each mouse was labeled with a specific number code (non-sequential), and then numbers were drawn randomly from a table to assign individual mice to various groups. The group allocation was masked until the interventions started. Group 1 was considered the control group and received normal saline (0.2 mL/mouse i.p.). Group 2 received MPTP (30 mg/kg/day i.p.) dissolved in saline for 5 days. Group 3 was given TUDCA (150 mg/kg/day i.p.) dissolved in phosphate-buffered saline (PBS) for 21 days following MPTP administration for 5 days (30 mg/kg/day i.p.). Group 4 received Syndopa (12 mg/kg/day p.o.) dissolved in filtered water for 21 days following MPTP administration for 5 days (30 mg/kg/day i.p.). Group 5 received TUDCA (150 mg/kg/day i.p.) and Syndopa (12 mg/kg day p.o.) for 21 days following MPTP administration (30 mg/kg/day i.p.) for 5 days. For additive/enhanced therapy, the time interval maintained each day between the TUDCA and Syndopa treatments for mice was 2 h.

### Cardiac perfusion and preservation of gut and midbrain tissues

At the end of the experimental procedures, an isoflurane inhalation anesthesia protocol was followed to perform a cardiac perfusion with normal saline (0.9% NaCl). The mice’s skulls were carefully opened to dissect the midbrain without mechanical damage. The midbrain was kept on ice and subsequently cleaned with ice-cold PBS, pH 7.4. A portion of the cut midbrain and gut tissues was fixed with 10% neutral buffered formalin, dehydrated, and then subjected to paraffin embedding and sectioning (5 µm thickness) for the purpose of immunohistochemical (IHC) analysis. While sampling the gut tissues, the distal colon region was processed for IHC staining. The technician who processed and stained the tissues for histological studies, as well as the pathologist who interpreted the slides, was blinded to the group identity at all stages of histological processing and quantification.

### Immunostaining of tyrosine hydroxylase (TH) protein expression in the striatum and SNpc

Slides containing midbrain sections were deparaffinized with washes in xylene for 5 min and 1-minute washes in a descending series of ethanol: 100%, 95%, 80%, and 70%. Antigen retrieval was performed with sodium citrate buffer. Briefly, 2.94 g of trisodium citrate dihydrate was dissolved in 95 mL of distilled water, and then the pH was adjusted to 6.0 using 1N NaOH to obtain the antigen retrieval buffer. Five mL of 0.1% Tween-20 was added and mixed well before adjusting the final volume to 100 mL with distilled water. After antigen retrieval, slides were kept in 5% hydrogen peroxide in methanol to quench endogenous peroxidase activity. Slides were washed in running tap water for 10 min, followed by 5 minutes in 0.1 M Tris, and then blocked in 3% fetal bovine serum (FBS) (SD Fine Chem, India). Slides were incubated in primary antibody overnight at 4^∘^C. The TH (Sigma-Aldrich) primary antibody was used at a 1:2000 dilution with 0.5%–1% formic acid (SRL Pvt Ltd, India). After washing the primary antibody with 0.1 M Tris for 5 minutes, incubation with goat anti-rabbit biotinylated IgG (Thermo Fisher Scientific, USA) at 1:1000 for 1 hour was performed. 0.1 M Tris buffer was used to rinse off the biotinylated antibody for 5 minutes, then the slides were incubated with avidin-biotin solution (Abcam, USA) for 1 hour. Slides were then rinsed for 5 minutes with 0.1 M Tris, and the sections were stained with diaminobenzidine (DAB) (SRL Pvt Ltd, India) to visualize the immune-positive cells. TH immune-positive areas were observed under a light binocular microscope (OLYMPUS CX23 Model).

### Immunohistochemical studies of NF-kb protein expression in the striatum and SNpc

The deparaffinization of slides with xylene and the washing protocol in a descending series of ethanol were detailed in the previous IHC methods section. After antigen retrieval (as described in the previous TH IHC methods section), slides were incubated in 0.3% hydrogen peroxide in methanol to quench endogenous peroxidase activity. Slides were blocked in PBS containing 0.2% Triton X-100 and 10% normal goat serum at 37 ^∘^C for 45 min. Slides were incubated at 4^∘^C in primary antibodies overnight. The rabbit anti-NFκB (Sigma-Aldrich) primary antibody was used at a 1:2000 dilution. After rinsing the primary antibody with 0.1 M Tris for 5 min, the sections were incubated with goat anti-rabbit biotinylated IgG at a 1:500 dilution at 37 ^∘^C for 45 min. The subsequent steps, such as washing the biotinylated antibody, incubation with avidin-biotin solution, rinsing with 0.1 M Tris, and the staining process with DAB to visualize the immune-positive cells, were followed exactly as indicated above in the TH IHC study protocol.

### IHC evaluation of α-synuclein protein expression in the gut and SNpc

For the evaluation of gut alpha-synuclein IHC expression, the transverse sections of the mucosa and submucosa of the colon were analyzed. The gut and midbrain sections were deparaffinized with 2 sequential 5-minute washes in xylene and 1-minute washes in a descending grade of ethanol: 100%, 100%, 95%, 80%, 70%. After antigen retrieval, slides were incubated in 5% H_2_O_2_ in methanol to quench endogenous peroxidase activity. Using running tap water, the slides were washed (10 min), in 0.1 M Tris (5 min), then blocked with 2% FBS. Incubation with the primary antibody was performed overnight at 4 ^∘^C. The anti-α-synuclein primary antibody (Abcam, USA) was used at a 1:1000 dilution. After rinsing off the primary antibody, the slides were treated with the secondary antibody (goat anti-rabbit biotinylated IgG) at a 1:1000 dilution for 1 hour. The remaining steps were followed exactly as detailed in the previous IHC methodology to visualize the α-synuclein positive immunoreactivity.

### Analysis and quantification of IHC slides

The TH, NFκB, and α-synuclein immunopositive areas detected in the target tissues were examined under a light binocular microscope (OLYMPUS CX23 Model), and the captured images were measured and quantified using ImageJ software version 1.54 (source: http://rsbweb.nih.gov/ij/download.html).

### Ethical statement

Animals were housed and well-maintained by adhering to the Committee for the Control and Supervision of Experiments on Animals (CCSEA), India, guidelines and protocols. The study procedures adhered to the IAEC guidelines and principles, and the research proposal was accepted by the Saveetha Medical College Institutional Animal Ethics Committee (SU/CLAR/RD/34/2023).

**Figure 1.**
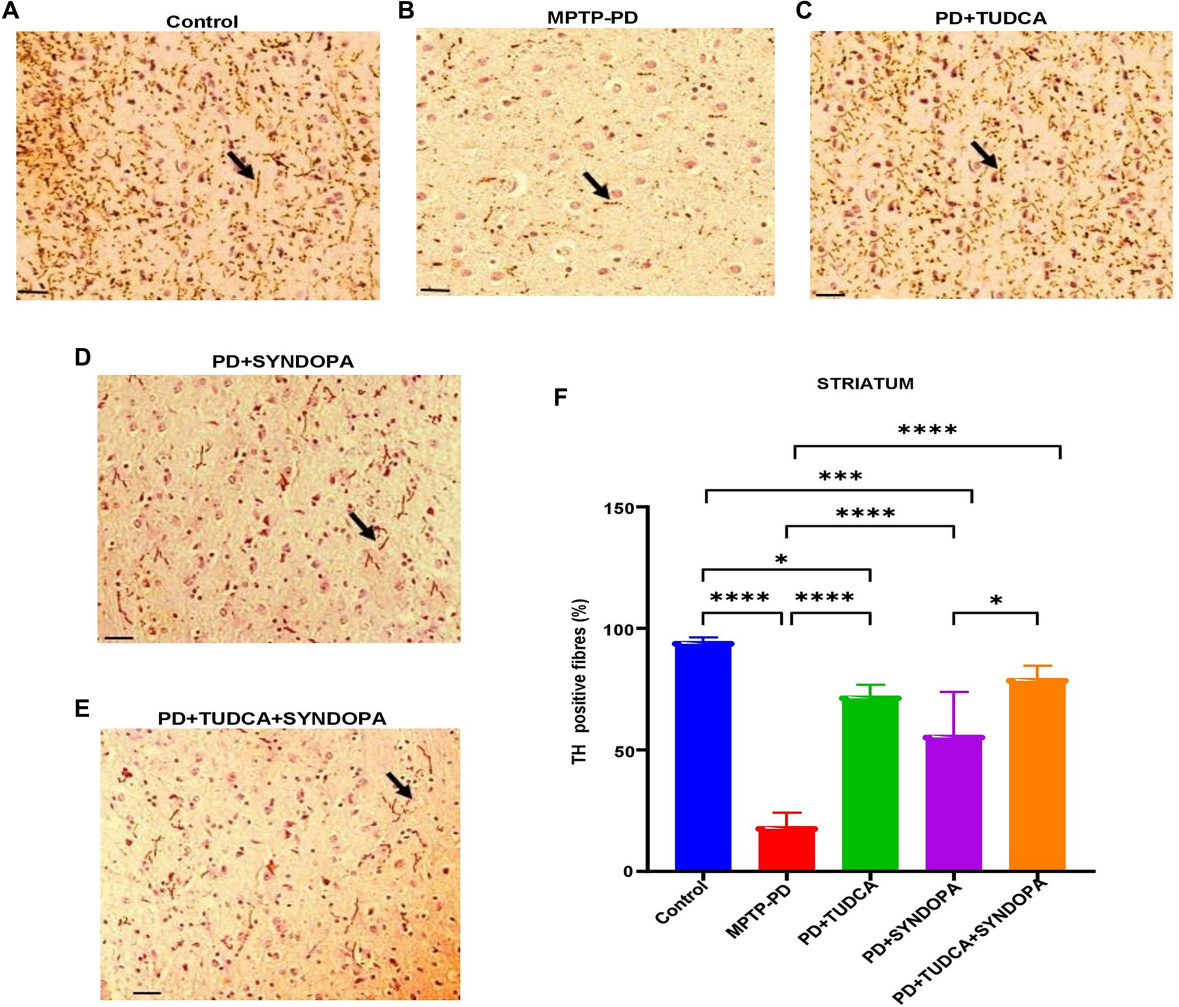
**Analysis of tyrosine hydroxylase expression in the brain striatum.** Midbrain sections from mouse brains were immunostained using a specific anti-tyrosine hydroxylase (TH) antibody. TH-positive fibers in the striatum were visualized under a light microscope and quantified. Panels (A−E) present representative microscopic images. Magnification: 400×; Scale bar: 20 µm. Panel F shows a bar graph of the percentage of TH-positive fibers in the brain striatum. Data are expressed as mean ± SD (*n* ═ 5 or 6). Statistical significance is indicated as *P < 0.05; ***P < 0.001; ****P < 0.0001.

### Statistical analysis

Statistical analysis was performed using GraphPad Prism software (GraphPad, La Jolla, CA, USA) with one-way ANOVA. Multiple comparisons were done using the Tukey test. The results are calculated as mean ± SD, and data are shown as mean + SD. *P* values of < 0.05 were considered statistically significant. In all the main figures, the statistically significant data are shown as ‘*’ according to the *P* values.

## Results

### Evaluation of TH protein expression in striatum and SNpc

The immunoreactivity of TH-positive fibers in the striatum (Figure 1 and Figure S1 with lower magnification-200x) of the control, MPTP-PD, TUDCA, Syndopa, and TUDCA + Syndopa groups were 94.6, 18.5, 72.2, 56.1, and 79.5%, respectively, and the differences were significant (*P* < 0.001). Compared to the control, the MPTP-PD and Syndopa groups showed significant differences in TH expression levels (*P* < 0.0001, and *P* < 0.05, respectively). By contrast, compared to the control group, the TUDCA and TUDCA + Syndopa groups did not show significant (*P* > 0.05) changes. The Syndopa group compared with the MPTP-PD group showed statistically significant changes (*P* < 0.001). When the Syndopa group was compared with the TUDCA monotherapy group, there was no statistical significance (*P* > 0.05). A similar trend was noted when the TUDCA group was compared with the TUDCA + Syndopa (*P* > 0.05). However, statistical significance exists when the TUDCA + Syndopa group was compared with the Syndopa group (*P* < 0.05).

**Figure 2.**
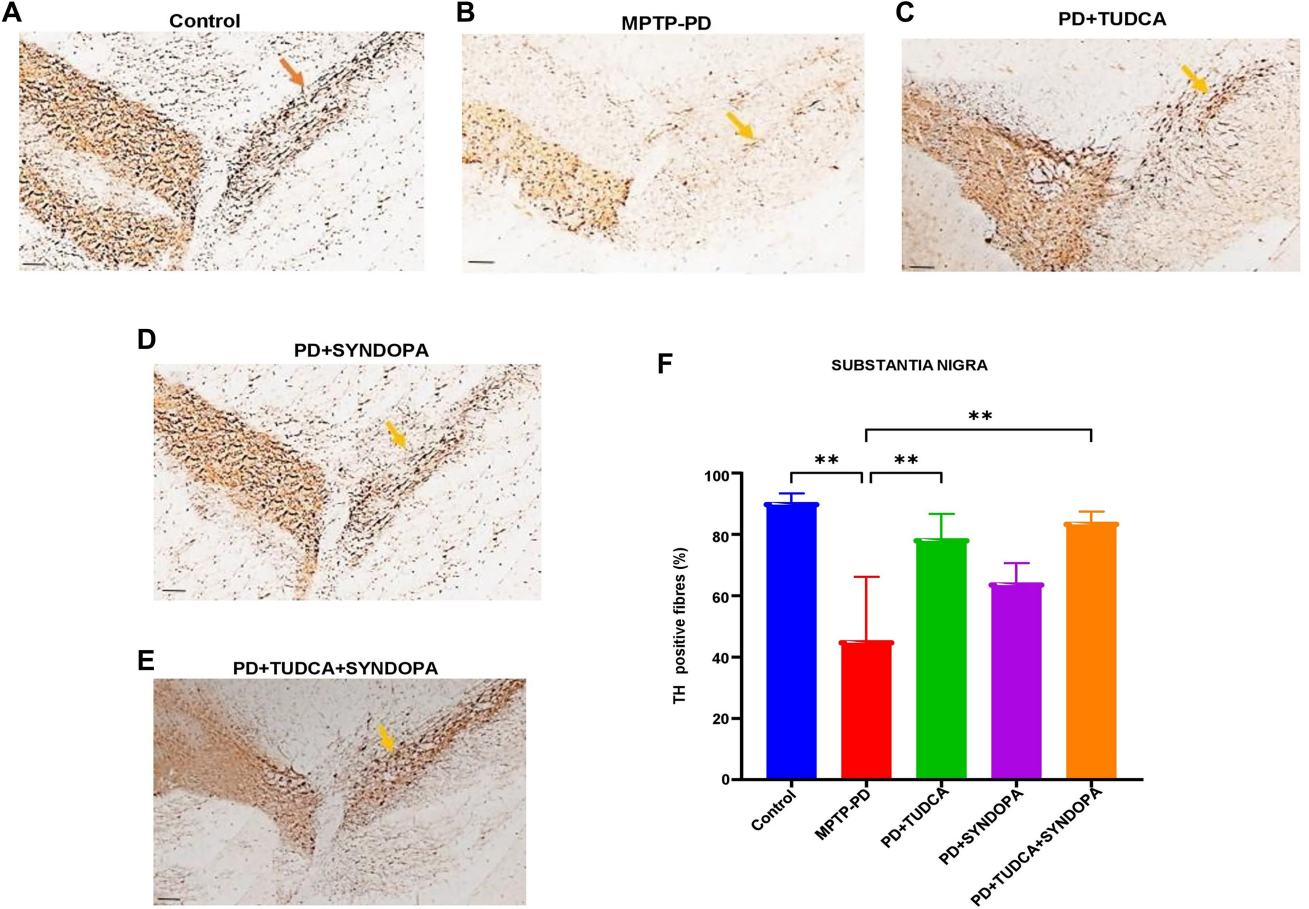
**Analysis of tyrosine hydroxylase expression in the substantia nigra pars compacta (SNpc).** Sections of mouse midbrain were immunostained using a specific anti-tyrosine hydroxylase (TH) antibody. TH-positive fibers in the SNpc were observed and quantified under a light microscope (panels A−E). Representative microscopic images are provided. Magnification: 200×; Scale bar: 200 µm. Panel F presents a bar graph depicting the percentage of TH fiber expression in the SNpc. Data are expressed as mean ± SD (*n* ═ 5 or 6). **P < 0.01.

The immunoreactivity of TH-positive fibers in the SNpc (Figure 2) of the control, MPTP-PD, TUDCA, Syndopa, and TUDCA + Syndopa groups was 90.4%, 45.3%, 78.6%, 64.1%, and 83.9%, respectively. Compared to the control group, the MPTP-PD group showed a significant decrease in TH protein expression levels (*P* < 0.01). Compared to the control group, the Syndopa group did not show significant differences (*P* > 0.05), and likewise, compared to the control group, the TUDCA group and TUDCA + Syndopa group also did not reveal significant differences in TH expression levels (*P* > 0.05). A comparison of the MPTP-PD group with either the TUDCA group or the TUDCA + Syndopa group revealed a statistically significant difference (*P* < 0.01). There was no significant difference when the Syndopa group was compared with the MPTP group (*P* > 0.05). When the Syndopa group was compared with either the TUDCA or TUDCA + Syndopa group, no statistical significance was observed (*P* > 0.05). Likewise, the comparison of the TUDCA group with the TUDCA + Syndopa group demonstrated no statistical significance (*P* > 0.05). However, the efficacy of TUDCA + Syndopa was marginally better than TUDCA monotherapy, whereas Syndopa alone treatment did not significantly improve the TH-positive fibers in the SNpc (*P* > 0.05).

### Evaluation of NF-kb protein expression in SNpc and striatum

**Figure 3.**
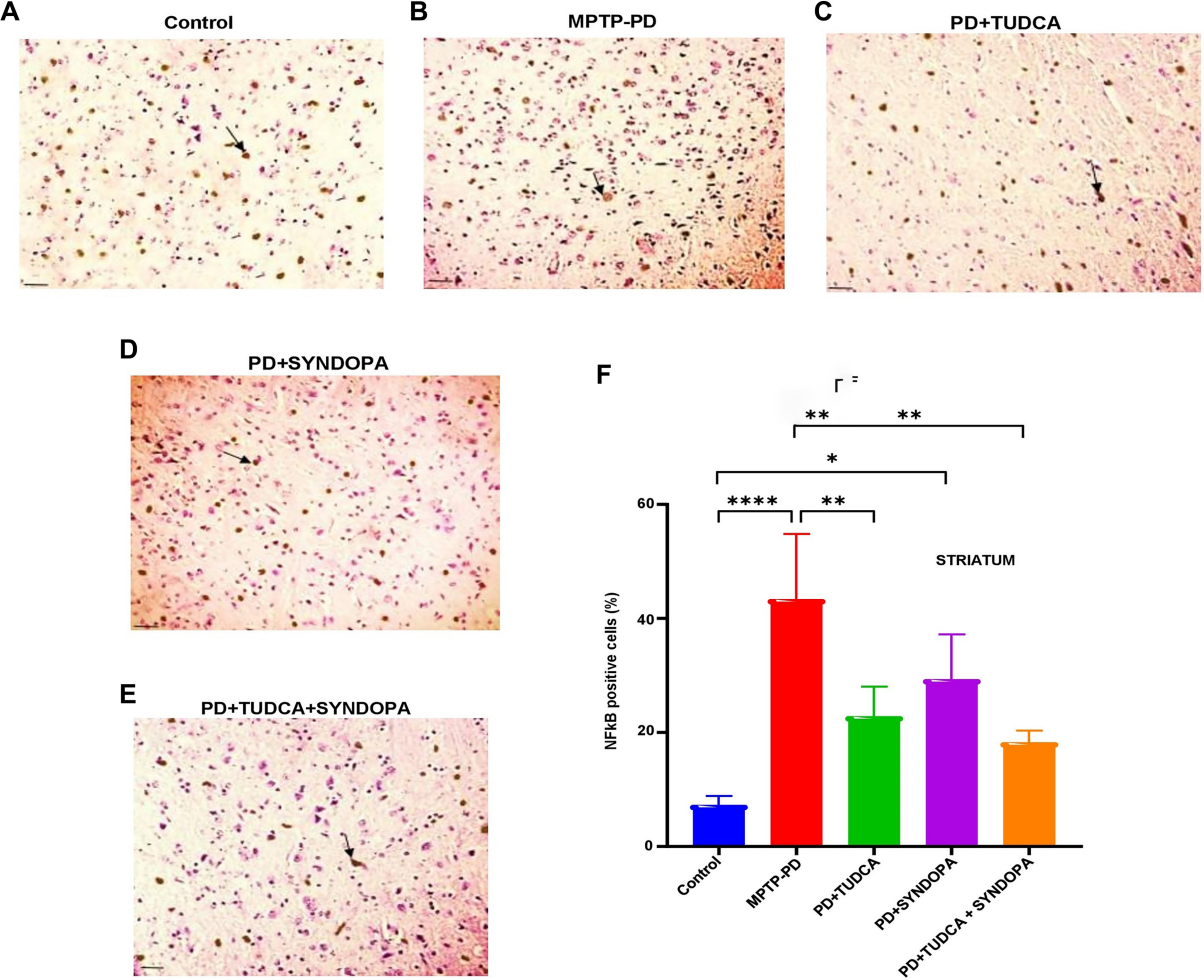
**Analysis of NF-kB expression in the brain striatum.** Midbrain sections of mouse brains were immunostained using a specific anti-NF-kB antibody. NF-kB-positive cells in the striatum were identified and quantified under a light microscope (A−E). Representative microscopic images are presented. Magnification: 600×; Scale bar: 50 µm. Panel F shows a bar graph of the percentage of NF-κB expression in the brain striatum. Data are expressed as mean ± SD (*n* ═ 5 or 6). *P < 0.05; **P < 0.01; ****P < 0.0001.

In the striatum (Figure 3 and Figure S2 with magnification 400×), the intensity of NF-kB-positive cells in the control, MPTP-PD, TUDCA, Syndopa, and TUDCA + Syndopa groups was estimated as 7.2%, 43.3%, 22.8%, 29.3%, and 18.2%, respectively. Compared to the control group, the MPTP-PD group showed significant differences in the NF-kB expression levels (*P* < 0.0001). Compared to the MPTP-PD group, the TUDCA group and TUDCA + Syndopa additive group exhibited significant differences in the expression levels (*P* < 0.01). A comparison made between the Syndopa group and the MPTP-PD group revealed no statistical significance (*P* > 0.05), although Syndopa treatment reasonably reduced NF-kB levels. There was no statistical significance (*P* > 0.05) when group comparisons were performed across all the drug-intervened groups (Syndopa vs TUDCA; Syndopa vs TUDCA + Syndopa; and TUDCA vs TUDCA + Syndopa). Overall, the NF-kB protein expression levels were significantly reduced in the TUDCA monotherapy group and the TUDCA plus Syndopa intervention group when compared with the MPTP-PD group.

**Figure 4.**
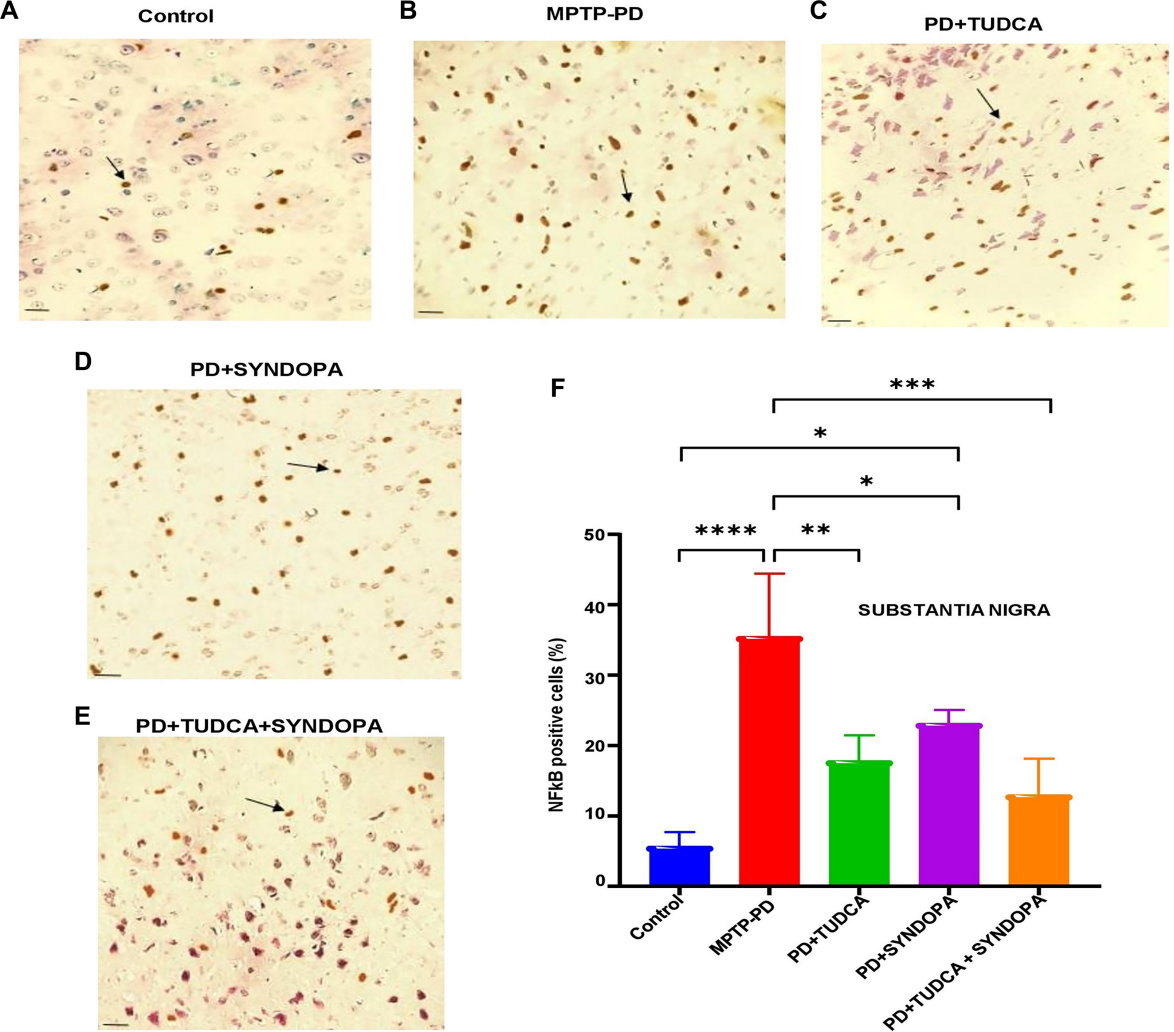
**Analysis of NF-kB expression in the substantia nigra pars compacta (SNpc) of the brain.** Mouse midbrain sections were immunostained using a specific anti-NF-kB antibody. NF-kB-positive cells within the SNpc were examined under a light microscope and quantified (A−E). Representative microscopic images are presented. Magnification: 600×; Scale bar: 50 µm. Panel F shows a bar graph of the percentage expression of NF-κB in the SNpc. Data are expressed as mean ± SD (*n* ═ 5 or 6). *P < 0.05; **P < 0.01; ***P < 0.001; ****P < 0.0001.

The intensity of NF-kB indicating cells in the SNpc (Figure 4 and Figure S3 with magnification 400×) of control, MPTP-PD, TUDCA, Syndopa, and TUDCA + Syndopa groups was shown as 5.70%, 35.48%, 17.82%, 23.15%, and 13.00%. Compared with control, MPTP-PD showed significant differences in the expression levels (*P* < 0.0001). In contrast, in various drug-treated groups (TUDCA, Syndopa, and TUDCA + Syndopa), the NF-kB expression levels were significantly reduced compared to the PD-MPTP group (*P* < 0.01, 0.05, and 0.001 respectively). As mentioned above for the striatum, statistical significance did not exist (*P* > 0.05) when the various drug intervention groups were compared with each other.

### Gut level changes in α-synuclein protein expression

**Figure 5.**
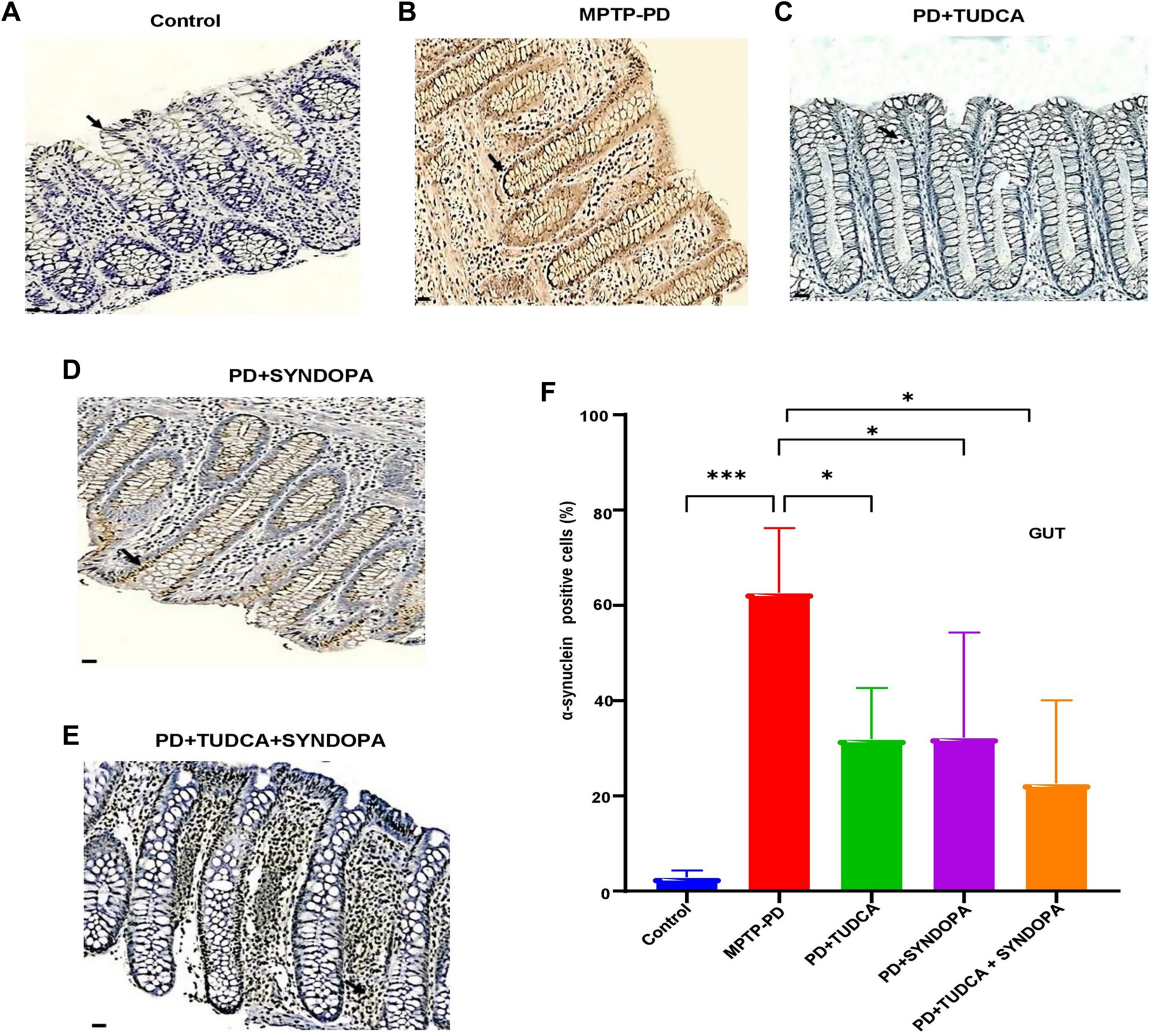
**Expression analysis of α-synuclein in the gut.** Transverse sections of mucosal and submucosal tissues were immunostained using a specific anti-α-synuclein antibody. α-Synuclein-positive cells in the gut tissues were examined under a light microscope and quantified (A−E). Representative microscopic images are displayed here. Magnification: 400×; Scale bar: 200 µm. Panel F shows a bar graph of the percentage expression of α-synuclein in mucosal and submucosal gut tissues. Data are expressed as mean ± SD (*n* ═ 5 or 6). *P < 0.05; ***P < 0.001.

The intensity of α-synuclein positive cells in gut samples (Figure 5 and Figure S4 with lower magnification 200×) of control, MPTP-PD, TUDCA, Syndopa, and TUDCA + Syndopa groups was manifested as 2.90%, 62.63%, 31.90%, 32.22%, and 22.56%. Compared with the control, the MPTP-PD group revealed significant upregulation of α-synuclein positive expression (*P* < 0.001). By contrast, Syndopa, TUDCA, and TUDCA + Syndopa treatments led to notable reductions in gut α-synuclein expression levels compared to the MPTP-PD group, and the values were found to be statistically significant (*P* < 0.05). The group comparisons made across the various drug treatment groups did not exhibit statistical significance (*P* > 0.05).

### Assessment of α-synuclein protein expression in SNpc

**Figure 6.**
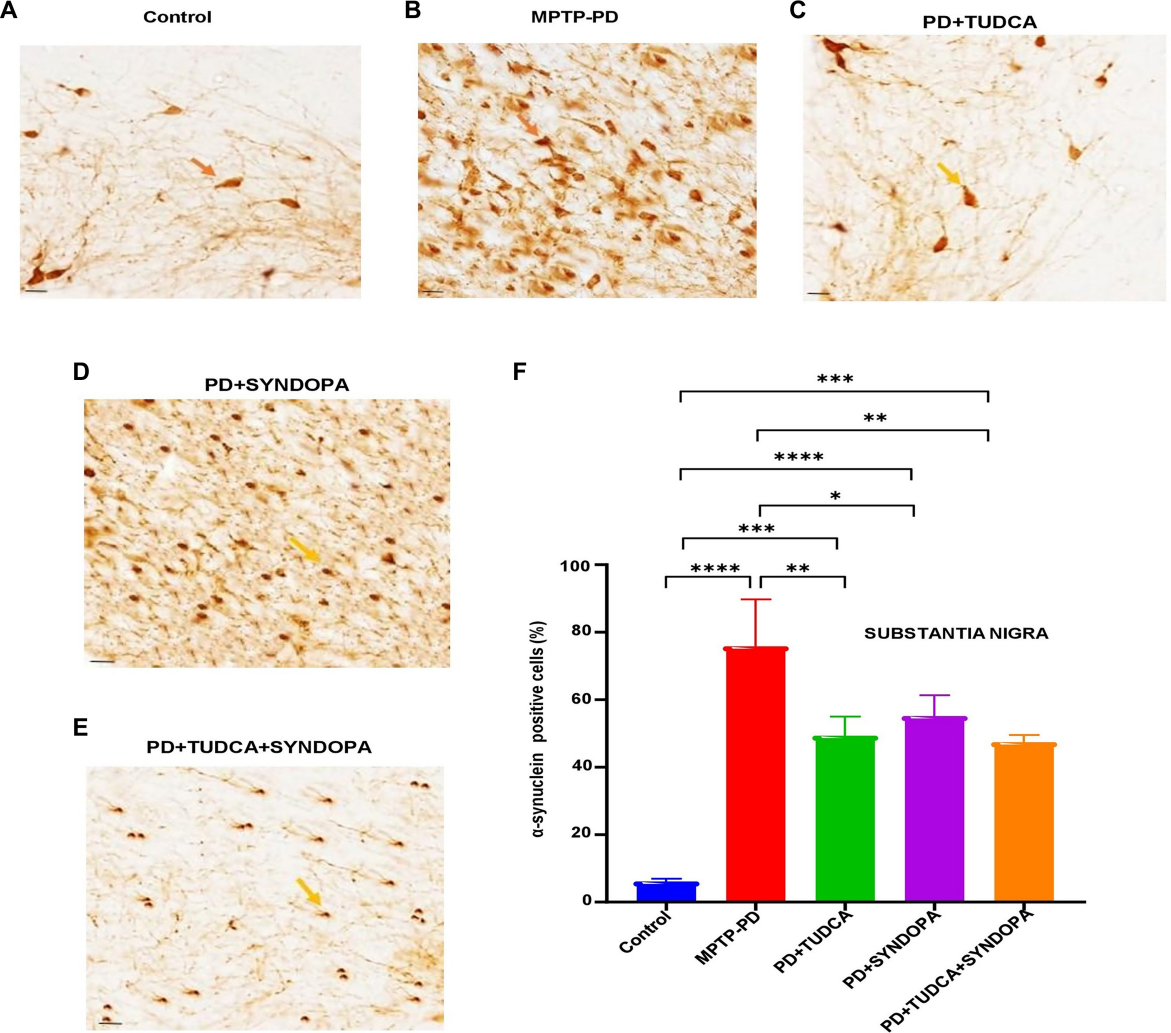
**Expression analysis of α-synuclein in the substantia nigra pars compacta (SNpc) of the brain.** Midbrain sections from mouse brains were immunostained using a specific anti-α-synuclein antibody. The α-synuclein-positive cells within the SNpc were examined under a light microscope and quantified. Panels (A−E) display representative microscopic images. Magnification: 400×; Scale bar: 50 µm. Panel F shows a bar graph of the percentage expression of α-synuclein in the SNpc. Data are expressed as mean ± SD (*n* ═ 5 or 6). Statistically significant results are indicated as follows: *P < 0.05; **P < 0.01; ***P < 0.001; ****P < 0.0001.

The intensity of α-synuclein positive cells (Figure 6 and Figure S5 with lower magnification 200×) in control, MPTP-PD, TUDCA, Syndopa, and TUDCA + Syndopa groups was found to be 6.06%, 75.80%, 49.28%, 55.08%, and 47.40%. Compared with the control group, MPTP-PD, TUDCA, Syndopa, and TUDCA + Syndopa showed strikingly elevated levels of α-synuclein (*P* < 0.0001, 0.001, 0.0001, and 0.001, respectively). TUDCA (*P* < 0.01), Syndopa (*P* < 0.05), and TUDCA + Syndopa (*P* < 0.01) groups also showed significant differences in the expression levels when compared with the MPTP-PD group, implicating the efficacies of drugs. There was no detectable level of statistical significance when the drug intervention groups were compared with each other (*P* > 0.05).

## Discussion

While PD is clinically diagnosed with cardinal motor symptoms that arise due to nigrostriatal degeneration, multiple pathologies coincide with PD pathogenesis. However, it remains unclear which pathology is initiated first to trigger nigrostriatal degeneration [35]. The current IHC data show prominent expression of α-synuclein in the gut samples of MPTP-intoxicated mice compared to the control group. Several notable studies have emphasized that misfolded α-synuclein is first formed in the enteric nerves before it can be detected in the brain. However, it is still intriguing whether PD starts first in the gut or the brain region. While many investigations provide evidence that MPTP administration increases alpha-synuclein expression in gut tissue and metabolic perturbation in the gut and gut dysbiosis [36, 37], the first and foremost region to be affected by MPTP is still under investigation. However, a recent publication by Heng et al. (2022) has mentioned that MPTP stimulates synucleinopathies first in the gut, contributing to the etiology and progression of PD. Heng et al. confirmed that synucleinopathies existed in the stomachs of both chronic and acute (single) 1-methyl-4-phenyl-1,2,3,6-tetrahydropyridine (MPTP)-injected mice. They first showed a significant elevation of aggregated and nitrated α-synuclein in the enteric glial cells (EGCs) of the gastric myenteric plexus. Next, they attempted to prove this mechanism by administering a single MPTP injection to mice. Stomach synucleinopathies were observed well before they could be visualized in the nigrostriatal system, specifically 12 h after MPTP injection. High MAO-B activity and low superoxide dismutase (SOD) activity in the stomach made it more susceptible to MPTP-induced oxidative stress, thus manifesting with increased reactive oxygen species (ROS) in the stomach and 4-hydroxynonenal (4-HNE) in the EGCs 3 h after MPTP exposure. Noticeably, a considerable increase in nitrated α-synuclein in the EGCs occurred after 3 h and 12 h of MPTP exposure. Taken together, the work of Heng et al. (2022) demonstrated that EGCs could be new contributors to synucleinopathies in the stomach. Therefore, it was reinforced that the early-initiated gut synucleinopathies may influence the adjacent neurons in the myenteric plexus and the CNS.

Based on epidemiological and histological findings, gastrointestinal problems (constipation) and α-synuclein inclusions were detected early in the ENS many years before the propagation of motor symptoms and inclusions in the CNS [38]. Another study depicted by Luk et al. (2012) [39] showed the ability of α-synuclein to undergo a “prion-like” misfolding and aggregation process, implicating that the disease may start in the peripheral organs such as the ENS and then progress to the CNS via the dorsal motor nucleus of the vagus, thereby affecting the brainstem, midbrain, and forebrain, and ultimately the cerebral cortex [38, 40].

The process by which misfolded α-synuclein present in the enteroendocrine cells (EECs) spreads from the gut epithelium to the brain was depicted by Haggerty et al. [41]. The same group has also shown how the neuron-like features of EECs connect to enteric nerves and take part in the dissemination of α-synuclein misfolding from the gut to the brain. The ability of TUDCA-Syndopa enhanced therapy in reducing gut α-synuclein levels is evident from the present IHC findings, which implicate amelioration of MPTP toxicity and early PD symptoms in mice.

While the loss of dopaminergic neurons is the primary cause of MPTP toxicity in the brain, a considerable number of research works have depicted that MPTP intoxication in mice and non-human primates can trigger α-synuclein expression in the SNpc [42–44]. In another report, a significant decrease (80%–89%) in TH-positive SNpc neurons on the side ipsilateral to MPTP administration in young and old MPTP-treated Rhesus monkeys was manifested, along with increased α-syn expression in the SN region [45]. Moreover, MPTP is still a widely used PD model for the preclinical testing of various pharmacological agents [46–48]. Although prolonged LD treatment could be responsible for the downregulation of TH, which is involved in dopamine synthesis, it may potentially lead to LD-induced dyskinesia. This could be the plausible reason that restoration of TH expression levels in the striatum by Syndopa is not as convincing as in other drug treatment groups. In the SNpc, TH expression was improved in the Syndopa group, although the value was not statistically significant in comparison with the MPTP group.

Neuroinflammation is an injurious pathological event in PD and AD. NF-κB, a key marker of neuroinflammation, is widely studied in PD models to explore both the pathological states of the disease and the efficacy of test drugs [49]. Neuroinflammation in the SNpc is greatly attributed to the progression of PD in mice [50]. NF-κB increased significantly in the striatum and SNpc of MPTP-induced mice [51, 52]. Using a mouse model of PD, it has been proven that drugs inhibiting NF-κB activation in the SNpc region effectively circumvent dopaminergic neuronal loss. So far, the anti-inflammatory potential of TUDCA in PD midbrains has been less studied; therefore, the present research involving an MPTP model of mice fills the research gap through IHC findings.

Decreased TH and increased α-synuclein expression in the midbrain and striatum have been depicted in MPTP and other chemical-induced PD models [53–55]. PD patients’ brain tissues have shown significantly reduced DA content based on several published works [56, 57]. Dopamine synthesis is triggered by TH via tyrosine hydroxylation to LD (precursor of dopamine). Importantly, the activity of TH in the brains of PD patients and PD mice has been shown to be significantly reduced [58–60]. TH+antibodies can detect the dopaminergic neurons in the SNpc pars compacta region in MPTP-induced mice [61]. The number of TH+ neurons in the SNpc was significantly reduced, with increased numbers of activated microglia in MPTP-treated mice [62]. Therefore, pharmacological approaches aimed at reducing TH levels in the striatum and SN regions in MPTP models were useful in reducing PD complications [63, 64]. It is evident that MPTP can activate glial cells in both the striatum and SNpc regions of the brain to alter proinflammatory markers, which are responsible for the promotion of neuroinflammation and neurodegeneration [65].

TUDCA intervention, either alone or administered along with Syndopa, reversed these changes considerably by reducing the damage caused by MPTP toxicity. The Syndopa treatment also had a better effect in counteracting MPTP toxicity, as shown in the present work. While striatal TH expression was significantly improved in the Syndopa treatment group, there was no significant restoration of TH in the SNpc region following Syndopa treatment. The region wise variations depicted by Syndopa seem interesting and need extensive investigation. The anti-inflammatory effect of Syndopa in the striatum is not pronounced in this MPTP model. All three drug intervention groups significantly inhibited α-synuclein accumulation in the gut tissues of MPTP-intoxicated mice, and the data is shown perhaps for the first time in the present study.

TUDCA crosses the BBB [66] to elicit a neuroprotective response. TUDCA’s anti-apoptotic mechanisms elicited in hepatocytes were found to also mimic those in neuronal cells [27–29]. Based on extensive literature evidence, it appears that bile acids can be used to defend against neuronal cellular programmed death pathways. Drugs that restored the loss of TH levels in the substantia nigra (SNpc) and striatal regions were found to be beneficial in ameliorating PD problems in MPTP, α-synuclein, and 6-hydroxydopamine models [67–70]. Protein oxidation, autophagy, and α-synuclein aggregation were attenuated by TUDCA when administered to mice before MPTP induction [71]. According to Rosa et al. [34], TUDCA pre-treatment of mice prevented MPTP toxicity by increasing the number of TH-positive cells in the striatum. By contrast, the present study supports the efficacy of TUDCA post-treatment in an MPTP model of PD. In addition, our current findings suggest the therapeutic potential of TUDCA and Syndopa additive therapy compared to TUDCA monotherapy for PD. Although the comparisons made across the various drug treatment groups were not significant in most of the study parameters, the mean values of the TUDCA + Syndopa group showed a better trend compared to the other drug groups tested.

Preclinical studies postulate that TUDCA’s therapeutic functions rely on anti-apoptotic and anti-neuroinflammatory mechanisms, apart from the suppression of oxidative stress and mitochondrial damage. From the present research findings, it is evident that TUDCA’s anti-inflammatory effect in the PD brain (both striatum and SNpc) is convincing when administered alone and is found to be better when Syndopa treatment is added. It also acts as a chaperone to sustain the stability and correct folding of proteins. Intriguingly, phase II clinical trials in AD have shown TUDCA to be a safe and potential drug. AD is one of the common neurodegenerative diseases that has tested hydrophilic bile acids as therapeutic agents. While much more clinical evidence is being gathered for other diseases, TUDCA holds promise for the treatment of neurodegenerative diseases [72]. Combinatorial therapy is gaining more attention in recent years for many neurological disorders, including PD [73–75]. Our earlier work, which tested embelin and levodopa combination therapy, provides additional proof of preclinical evidence to substantiate the importance and efficacy of combined therapy in the amelioration of PD complications [24, 76].

Study Limitations: (i) The present study did not test the behavioral parameters (functional activity) to assess the additive effects of TUDCA and Syndopa treatments in PD model mice. Therefore, future work relies on these preclinical data to validate the drug efficacies and to ensure the potential for a cure in translational research; (ii) the validity of the MPTP model is not high, and this model does not feature all the problems/symptoms of PD patients; and (iii) it seems likely that antioxidant monotherapy is insufficient for disease modification in humans.

## Supplemental data

Supplemental data are available at the following link: https://www.bjbms.org/ojs/index.php/bjbms/article/view/12519/3979.

## Data Availability

The complete data sets in support of this work is available with the Corresponding author and will be shared upon request.
